# Development of Indicator Film Based on Cassava Starch–Chitosan Incorporated with Red Dragon Fruit Peel Anthocyanins–Gambier Catechins to Detect Banana Ripeness

**DOI:** 10.3390/polym15173609

**Published:** 2023-08-31

**Authors:** Valentia Rossely Santoso, Rianita Pramitasari, Daru Seto Bagus Anugrah

**Affiliations:** 1 Food Technology Study Program, Faculty of Biotechnology, Atma Jaya Catholic University of Indonesia, BSD Campus, Tangerang 15345, Indonesia; valentiarossely@gmail.com; 2 Biotechnology Study Program, Faculty of Biotechnology, Atma Jaya Catholic University of Indonesia, BSD Campus, Tangerang 15345, Indonesia; daru.seto@atmajaya.ac.id

**Keywords:** red dragon fruit peel anthocyanins, gambier catechins, indicator film, co-pigmentation, banana ripeness

## Abstract

Banana ripeness is generally determined based on physical attributes, such as skin color; however, it is considered subjective because it depends on individual factors and lighting conditions. In addition, improper handling can cause mechanical damage to the fruit. Intelligent packaging in the form of indicator film incorporated with anthocyanins from red dragon fruit peel has been applied for shrimp freshness detection; however, this film has low color stability during storage, necessitating the addition of gambier catechins as a co-pigment to increase anthocyanin stability. Nevertheless, the characteristics of films that contain gambier catechins and their applications to bananas have not been studied yet; therefore, this study aims to develop and characterize indicator films that were incorporated with red dragon fruit peel anthocyanins and gambier catechins to detect banana ripeness. In this study, the indicator films were made via solvent casting. The films were characterized for their structural, mechanical, and physicochemical properties, and then applied to banana packaging. The results show that the film incorporated with anthocyanins and catechins in a ratio of 1:40 (*w*/*w*) resulted in better color stability, mechanical properties, light and water vapor barrier ability, and antioxidant activity. The application of the indicator films to banana packaging resulted in a change in color on the third day of storage. It can be concluded that these films could potentially be used as an indicator to monitor banana ripeness.

## 1. Introduction

Bananas are Indonesia’s leading horticultural product. In 2022, Indonesia produced 9,245,427 tons of bananas, which were then marketed both globally and domestically [1]. The distribution of bananas for retail marketing is carried out when the banana reaches a certain level of maturity according to a reference color chart, with a score of 3 indicating a banana that is green–yellow, a score of 4 indicating a banana that is green and more yellow, and a score of 5 indicating a banana that is yellow [2]. The maturity level of a banana is generally determined by comparing the color of the fruit skin with a reference color chart, but this method is considered to be very subjective and inconsistent because it depends on operator skill and lighting [3]. In addition, improper handling of the fruit when determining ripeness can cause mechanical damage [4].

Intelligent packaging is a type of food packaging that can detect and provide information about the quality of food ingredients in such aspects as freshness, ripeness, gas production, and the presence of microbial contamination in the ingredient [5]. Intelligent packaging is generally equipped with sensor technology, enabling the collection of information without damaging or opening the package. Sensors that are commonly used in intelligent packaging are color indicators, which are incorporated into polymer-based films and are sensitive to pH, allowing for a color change when there is a change in the pH of the product [6]. 

Anthocyanins are water-soluble natural pigments that originate from red-, blue-, purple-, and black-colored materials, such as red dragon fruit [7], butterfly pea flower [8], purple sweet potato [9], and black rice [10]. These pigments can change color based on changes in the pH of their environment. This characteristic has led to the widespread development of anthocyanins as color indicators incorporated into films made from materials such as whey protein isolate nanofibers [11], chitosan [12], alginate [13], gelatin [14], and pectin–chitosan [10], primarily to monitor the freshness of meat and seafood. Additionally, anthocyanins incorporated into films can also be utilized to determine the maturity levels of fruits, including bananas. Bananas are climacteric fruits, where the respiration process will continue even beyond the harvest process. The fruit ripening process causes an increase in the pH, which can be used as an indicator of the level of fruit maturity [5].

Research on indicator films based on cassava–chitosan starch with anthocyanins from red dragon fruit peel has been carried out to detect freshness in shrimp; however, it produces less stable colors during storage [15]. Anthocyanin-based indicator films are susceptible to light and temperature. Light and temperature cause discoloration on the films even when they are not used for food monitoring [16]. One method that can be applied to increase the color stability of anthocyanins is co-pigmentation. Co-pigmentation of anthocyanins using gambier catechins (*Uncaria gambier*) has been carried out and proven to minimize the degradation of black rice anthocyanins in isotonic drinks [17]; however, improvements in the color stability of anthocyanin-based indicator films with gambier catechin co-pigmentation have never been carried out.

In order to fill this research gap, this study aimed to develop and characterize indicator films based on a cassava starch–chitosan complex and anthocyanins of red dragon fruit (*Hylocereus polyrhizus*) peel co-pigmented with gambier catechins as indicators to monitor banana ripeness. The success of the film’s development can be understood based on the film’s ability to detect the maturity level of bananas as climacteric fruits that produce volatile compounds that increase the pH in the environment inside the package.

## 2. Materials and Methods

### 2.1. Materials

Red dragon fruit, cavendish banana (*Musa acuminata* L.), and tapioca flour (Rose Brand) were obtained from AEON Mall BSD City, Tangerang, Indonesia. Chitosan with a deacetylation degree of 83.3% and molecular weight of 70 kDa was obtained from Xi’an Gawen, China. Ethanol, glacial acetic acid, and 2,2-diphenyl-1-picrylhydrazyl (DPPH) were obtained from Smart-Lab, Tangerang, Indonesia. Glycerol was obtained from Merck KGaA, Darmstadt Germany. The other ingredients included gambier catechins and distilled water. All chemicals used in this research were analytical grade.

### 2.2. Methods

#### 2.2.1. Anthocyanin Extraction from Red Dragon Fruit Peel

The red dragon fruit was washed, and the flesh was removed. Then, the red dragon fruit peel was mashed using a food processor (Philips 2061, Shanghai, China) until it became mushy. Once smooth, 100 g of red dragon fruit peel pulp were weighed and placed into 500 mL of a 70% ethanol solution. Then, the mixture was stirred using a stir bar until the mixture became evenly distributed. The mixture was then macerated in a chiller at 5 °C for 24 h. After maceration, the extract was filtered using Whatman No. 1 paper. The filtrate obtained was then concentrated using a rotary evaporator (R-300 Buchi, Flawil, Switzerland) at 55 °C, 60 rpm speed, and 130 mbar pressure [18].

#### 2.2.2. Analysis of the Anthocyanin Levels of Red Dragon Fruit Peel

Every 100 µL of the concentrated anthocyanin extract was mixed into 1.9 mL of a potassium chloride buffer solution (pH 1) and a sodium acetate buffer solution (pH 4.5) in a test tube. Next, the solution was homogenized using a vortex (Thermo Fisher, Waltham, MA, USA) and incubated for 15 min. After that, the absorbance of the solution was measured at wavelengths of 536 and 700 nm using a UV-Vis spectrophotometer (Thermo Scientific, Waltham, MA, USA). The blank used in this measurement was distilled water. The total anthocyanin level was calculated using the following equation [19]:

(1) $$\mathrm{Anthocyanin}\;\mathrm{level}\;(\mathrm{mg}/L)=\frac{A\times MW\times DF\times 1000}{\varepsilon \times L}$$

where *A* is the absorbance value, with *A* = (*A*_536_ − *A*_700_) pH 1 − (*A*_536_ − *A*_700_) pH 4.5; *MW* is the molecular weight of anthocyanin (449.2 g/mol); *DF* is the dilution factor; ε is the molar attenuation coefficient of anthocyanin (26,900 L/mol·cm); and L is the width of the cuvette (1 cm).

#### 2.2.3. Addition of Gambier Catechins

Gambier catechins were added to the anthocyanin extract of the red dragon fruit peel following four discrete formulations, as seen in Table 1 [20].

The color extract was prepared by dissolving gambier catechin powder in concentrated anthocyanin extract, which was then homogenized using a magnetic hotplate stirrer (Heidolph MR Hei-Standard, Schwabach, Germany) for 30 min. The solution was then filtered using Whatman No. 1 paper to obtain the filtrate.

#### 2.2.4. Color Change Analysis of the Color Extracts

A total of 13 cuvettes were prepared. Then, 1 mL of color extract was placed into each cuvette and 1 mL of pH 1–13 buffer was added to each cuvette, so that a final volume of 2 mL was obtained. The solution was then incubated for 30 min, and then the color change was observed and photographed [21].

#### 2.2.5. Indicator Film Preparation

The indicator films were prepared by first preparing a 1% (*w*/*v*) chitosan solution involving the mixing of 1 g of chitosan into 100 mL of 1% (*v*/*v*) acetic acid solution. The solution was homogenized using a magnetic hotplate stirrer (Thermo Scientific, Waltham, MA, USA), with stirring at 300 rpm for 24 h. Next, a 2% (*w*/*v*) cassava starch solution was prepared by mixing 2 g of cassava starch powder into 100 mL of distilled water. The mixture was then gelatinized using a magnetic hotplate stirrer (Thermo Scientific, Waltham, MA, USA) until it reached a temperature of 80 °C.

The chitosan solution was then mixed with the gelatinized cassava starch solution in a ratio of 1:1. After mixing, the solution was then homogenized using a homogenizer (Heidolph, Schwabach, Germany) at 530 rpm for 10 min. Then, 85% glycerol was added to the solution, and homogenization was performed using a homogenizer (IKA T10 ultra-turrax, Potsdam, Germany) at 14,500 rpm for 5 min (Table 2). The color extract was then added to the film solution and stirred using a stir bar until it became smooth [22].

#### 2.2.6. Indicator Film Casting

Each variation of the film solution was poured into a plastic Petri dish 6 cm in diameter at 20 mL. Next, the solution was dried in a fume cupboard for 72 h. After the solution dried and formed a film, the film was removed from the Petri dish using a spatula and stored in a closed, dark container at a chiller temperature of 5 °C [23].

#### 2.2.7. Characterization of Indicator Films’ Surface Color

The surface colors of the four indicator film samples were analyzed using a colorimeter (Nh3, China) on the 1st and 14th day of storage at a temperature of 5 °C. After obtaining the *L*, *a*, and *b* values from the film samples, the total color difference (∆*E*) was calculated using the following formula [21]:

(2) $$\Delta E=\sqrt{{\left(L_{s}-L\right)}^{2}+{\left(a_{s}-a\right)}^{2}+{\left(b_{s}-b\right)}^{2}}$$

where *L* is lightness, *a* is red (+) and green (−), and *b* is yellow (+) and blue (−).

#### 2.2.8. Analysis of Indicator Film Sensitivity to Various pH Conditions

The films’ sensitivity to pH and their color-changing ability were analyzed. The indicator film was cut to a size of 1 × 1 cm and then dipped in each buffer solution with a pH value of 1–13 for 2 min, respectively [21].

#### 2.2.9. Characterization of Indicator Film Structure

The surface morphology of the films was observed using scanning electron microscopy (SEM) (Hitachi SU3500, Tokyo, Japan) with an electric voltage of 10 kV and high vacuum conditions (10^−3^). An FTIR analysis in the range of 500–4000 cm^−1^ (Bruker-Tensor II, Borken, Germany) was performed to observe the functional groups on the films [24].

#### 2.2.10. Characterization of Mechanical Properties of the Films

The film thickness was measured using a micrometer with an accuracy of 0.01 mm. The mechanical properties were then analyzed using a texture analyzer (Agrosta Texturometer, Serqueux, France). The film samples were cut to a size of 4 × 1 cm and affixed to the towing lever. A tensile test was then carried out with an initial grip of 1 cm and a cross-head speed of 0.8 mm/s. The tensile strength and elongation at break, as measurements of film strength and flexibility, were determined using the following formula [21]:

(3) $$Tensile\;strength\;\left(MPa\right)=\frac{F}{A_{0}}\;$$

(4) $$Elongation\;at\;break\;\left(\%\right)=\frac{\delta \;}{L_{0}}\times 100$$

where *F* is the force applied to the film sample, $A_{0}\;$is the area of the film sample, δ is the increase in length, and $L_{0}\;$is the initial length of the film sample.

#### 2.2.11. Physical Characterization of Films

##### Analysis of Transparency and Transmittance of the Indicator Films

The films were cut to a size of 4 × 1 cm and affixed to the clear side of the cuvette. The light transmittance of the films could be measured using a UV-Vis spectrophotometer (Shimadzu UV-2450, Tokyo, Japan) by measuring the transmittance at a wavelength of 200–800 nm. After determining the percentage of light transmittance, the transparency of the film was calculated using the following formula [21]:

(5) $$Transparency=\frac{logT600}{D}\;\;$$

where *T*600 is the percent transmittance of light at a wavelength of 600 nm and *D* is the thickness of the film sample (mm).

##### Analysis of Moisture Content and Solubility of the Indicator Films

Analyses of the film water content and solubility were carried out based on the method of Alizadeh-Sani et al. [21]. The films were cut to a size of 2 × 2 cm. The moisture content of the film samples was then measured by weighing the film samples before drying (*W*1) and after drying (*W*2) using an oven (Memmert UN110, Greifensee, Switzerland) at 105 °C for 24 h using the following formula [21]:

(6) $$Moisture\;content\;\left(\%\right)=\frac{W1-W2}{W1}\times 100$$

For the film solubility analysis, 2 × 2 cm films were dried in an oven (Memmert UN110, Greifensee, Switzerland) at 105 °C for 24 h and then weighed as *W*1. The dried film samples were immersed in 25 mL of distilled water involving shaking using an orbital shaker (GFL 3017, Greifensee, Germany) at 100 rpm for 24 h. The film samples were then dried again using an oven (Memmert UN110, Greifensee, Switzerland) at 105 °C for 24 h and then weighed as *W*2. After weighing, the solubility of the film samples was calculated using the following formula [21]:

(7) $$Water\;solubility\;\left(\%\right)=\frac{W1-W2}{W1}\times 100$$

##### Analysis of Water Vapor Transmission Rate (*WVTR*)

The *WVTR* value was calculated by cutting a circular film sample 4 cm in diameter. The cut film was then used as a lid for a container containing 5 g of silica gel (0% RT), which was placed in a desiccator filled with distilled water (99% RH). The silica gel was weighed and replaced periodically every 24 h for 3 days. From the results, a graph of the mass of the container against time was obtained and a linear equation was developed using the formula y = mx + c. Then, the *WVTR* value was calculated using the following formula [21]:

(8) $$WVTR=\frac{m}{A\times t}\;$$

The *WVTR* value obtained is in units of gm^−2^·24 h^−1^. The value of m is obtained from the linear equation, *A* is the film area (m^2^), and t is the time (hours).

#### 2.2.12. Analysis of Antioxidant Activity

As much as 50 mg of each film sample was weighed and dissolved in 5 mL of distilled water and homogenized using a vortex (Thermo Fisher, Waltham, MA, USA). The solution was then shaken using an orbital shaker (GFL 3017, Burgwedel, Germany) at 200 rpm for 24 h. The extract from the film was then taken and used for testing using the radical scavenging 2,2-diphenyl-1-picrylhydrazyl (DPPH) method. The test solution was prepared by dissolving 2.5 mg of DPPH in 100 mL of 99.7% methanol, with distilled water as a blank. A total of 4 µL of extract from the film was mixed with 196 µL of DPPH solution, then the mixture was homogenized using a vortex (Thermo Fisher, Waltham, MA, USA). The absorbance was measured at a wavelength of 490 nm using a microplate reader (NanoQuant TECAN, Hombrechtikon, Switzerland). The DPPH activity was then calculated using the following formula [25]:

(9) $$Antioxidant\;activity\;\left(\%\right)=\frac{A_{DPPH}-A_{film}\;}{A_{DPPH}}\times 100$$

*A_DPPH_* is the absorbance of the blank DPPH in the form of a mixture of DPPH solution and distilled water, while *A_film_* is the absorbance of the DPPH film sample.

##### Monitoring the Ripeness of Bananas

Each indicator film sample was cut to a size of 1.5 × 1.5 cm and then affixed to PVC cling wrap, which was part of the lid of the container. The film was not allowed to come into direct contact with the banana sample. The prepared samples were stored at room temperature for 7 days. The color changes of the films were observed and photographed every day [26].

##### Statistical Analysis

The experiments in film-making and film characterization were repeated three times. The data obtained were then statistically processed using the IBM SPSS Statistics 25 (IBM Corp., Armonk, NY, USA) application. First of all, a normality test was performed on the data using the Shapiro-Wilk test. If the data were normally distributed, then the data were tested for homogeneity. If the data were normally distributed and homogeneous, then the test was followed by a one-way analysis of variance (ANOVA). The real differences obtained were analyzed using Duncan’s test at α = 0.05. If the data were otherwise not normally distributed and/or not homogeneous, then the data were processed using the Kruskal–Wallis test, followed by the stepwise step-down test [27].

## 3. Results

### 3.1. Anthocyanin Level of Red Dragon Fruit Peel Extract

**Figure 1:**
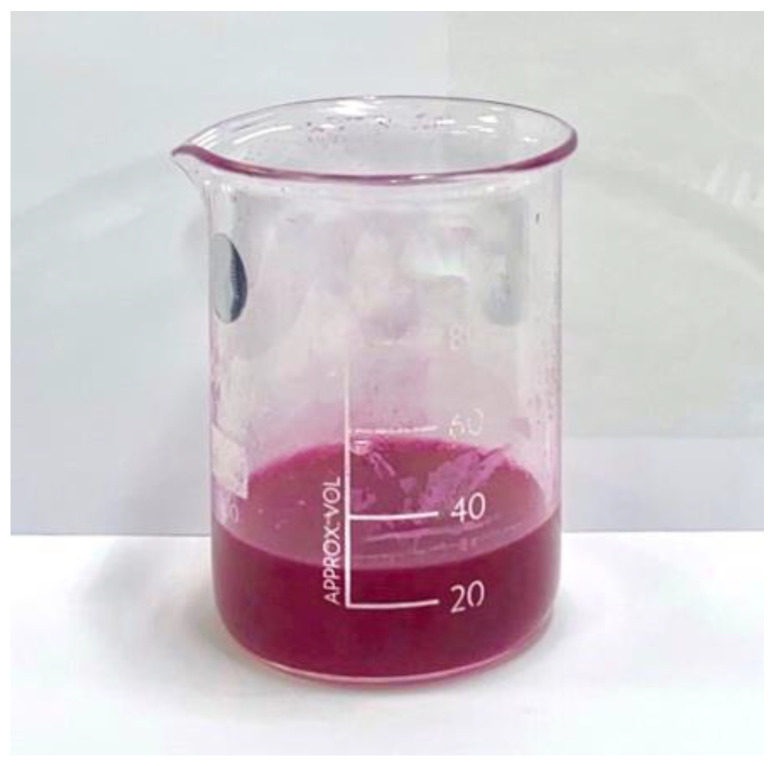
Anthocyanin extract of red dragon fruit peel.

The anthocyanin extract of red dragon fruit peel obtained was purplish-red in color (Figure 1). The extraction of anthocyanin by the maceration method using 70% ethanol resulted in an anthocyanin level of 0.122 ± 0.01 mg/g.

### 3.2. Changes in Extract Color under Various pH Conditions

**Figure 2:**
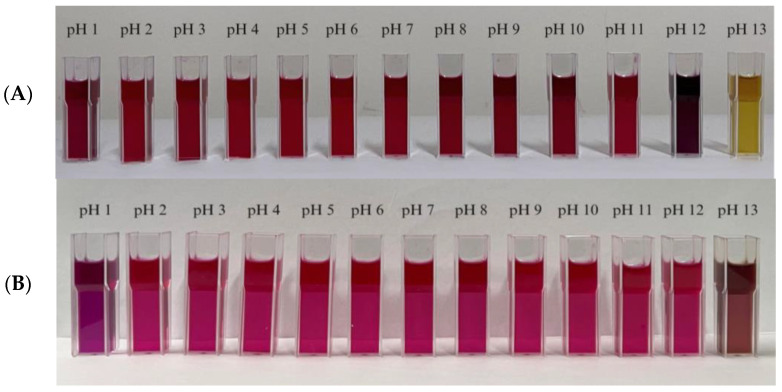
Color change test of the color extract (**A**) before the addition of gambier catechin and (**B**) after the addition of gambier catechin.

The color extract was divided into two categories, namely, anthocyanin extract from red dragon fruit peel without the addition of gambier catechin and anthocyanin extract from red dragon fruit peel with the addition of gambier catechin. In the color extract without the addition of gambier catechin, the color changed to purple–yellow at a pH of 12 and to yellow at a pH of 13. In the color extract with the addition of gambier catechin, color changes appeared at pH 1 to purple and at pH 13 to yellowish-brown (Figure 2).

### 3.3. Indicator Films

**Figure 3:**
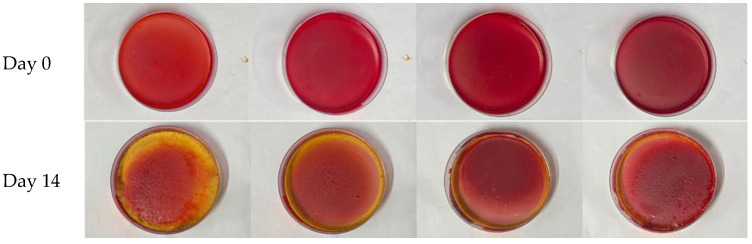
The color changes of the indicator films (SCh–A (cassava starch–chitosan, glycerol, anthocyanin), SCh–AC20 (cassava starch–chitosan, glycerol, anthocyanin–catechin 1:20), SCh–AC28 (cassava starch–chitosan, glycerol, anthocyanin–catechin 1:28), and SCh–AC40 (cassava starch–chitosan, glycerol, anthocyanin–catechin 1:40) after 14 days of storage.

The final results of the film-making and the condition of each film after 14 days of storage can be seen in Figure 3. During the 14 days of storage, it was clear that the SCh–A and SCh–AC20 films had changed in color from pink to red–yellow, whereas the SCh–A, SCh–AC28, and SCh–AC40 films had only a slightly yellowish tint to the outside of the films.

### 3.4. Surface Colors of the Indicator Films

After 14 days of storage, the SCh–A film showed increased L*, a*, and b* values. The SCh–AC20 film showed increased L* and b* values, but the a* value decreased. The SCh–AC28 film showed decreased L*, a*, and b* values. Meanwhile, the SCh–AC40 film showed decreased L* and a* values, but the b* value increased. This indicates that the color of the film grew darker and yellower, and the redness decreased toward green. Based on the L*, a*, and b* values, the SCh–AC40 film had the lowest color change. This is indicated by the smaller Δ*E* value of the SCh–AC40 film compared to the other film samples (Table 3).

### 3.5. Indicator Film Sensitivity to Various pH Conditions

**Figure 4:**
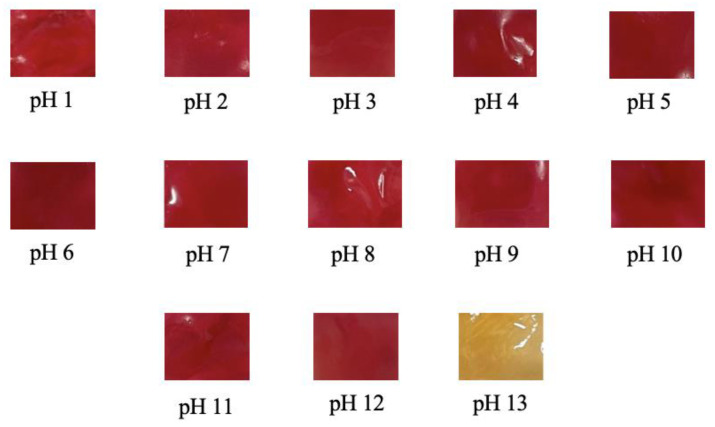
The SCh-AC40 indicator film’s sensitivity to various pH conditions.

The sensitivity of an indicator film to various pH conditions was tested only in SCh–A40 because of the film’s smallest Δ*E* value. The results of the evaluation of the SCh–AC40 indicator film that was dipped in pH 1–13 buffer can be seen in Figure 4. It was discovered that the indicator film had the ability to change color according to the pH conditions from red at pH 1–12 to yellow at pH 13.

### 3.6. Structural Properties of the Indicator Films

**Figure 5:**
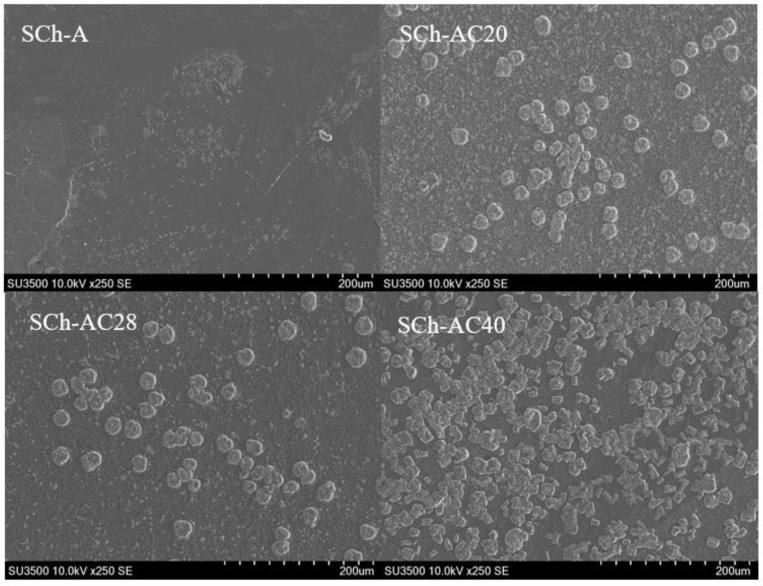
Indicator film morphology.

The morphology of the indicator films observed using SEM can be seen in Figure 5. The SCh–AC40 film showed very clear gray and white aggregates. As the amount of gambier catechins added increased, the number of aggregates captured by SEM increased as well.

**Figure 6:**
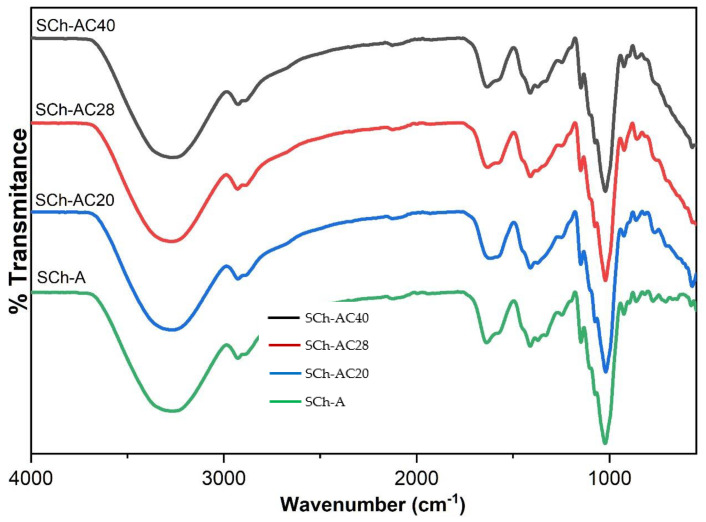
FTIR results of the indicator films.

The results of the FTIR curve analysis can be seen in Figure 6. The spectrum produced by each film was similar, with differences found only in the absorption at 500–1000 cm^−1^, which was caused by differences in the stretching of C–O due to the addition of the gambier catechins.

### 3.7. Thickness and Mechanical Properties of the Indicator Films

The SCh–AC20 and SCh–AC28 films showed the highest thickness values. Based on the mechanical properties results, the SCh–AC40 film had the highest tensile strength and elongation at break values, while the SCh–AC20 film had the lowest tensile strength and elongation at break values (Table 4).

### 3.8. Physical Properties of the Indicator Films

#### 3.8.1. Transmittance and Transparency of the Indicator Films

**Figure 7:**
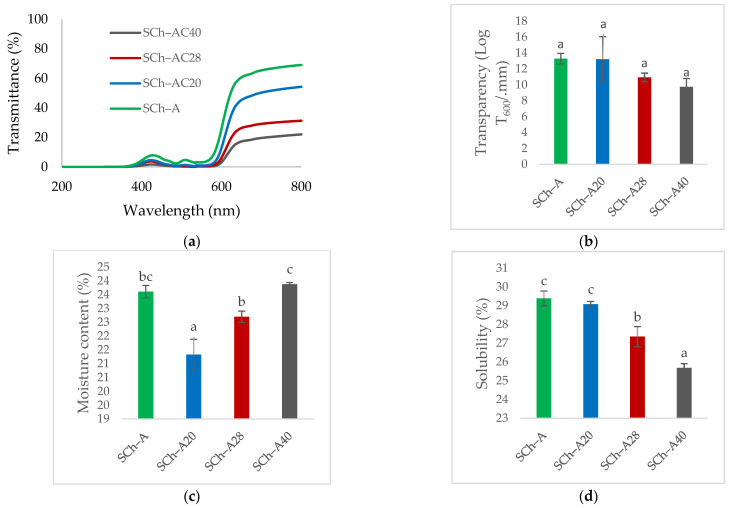

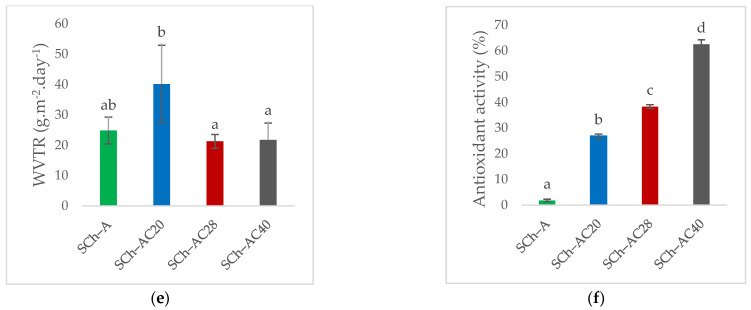
Physicochemical properties of SCh-A, SCh-A20, SCh-A28, and SCh-A40 indicator films through (**a**) transmittance; (**b**) transparency; (**c**) moisture content; (**d**) solubility; (**e**) water vapor transmission rate (WVTR); and (**f**) antioxidant activity. Bars represent means ± standard deviations. Different letters indicate a real difference; α ≤ 0.05.

The light transmittance value of each film increased at wavelengths of 380 nm and 570 nm; however, it experienced a decrease at a wavelength of 450 nm (Figure 7a).

The results for the transparency of the indicator films can be seen in Figure 7b. The transparency values obtained for the SCh–A, SCh–AC20, SCh–AC28, and SCh–AC40 films were not significantly different.

#### 3.8.2. Moisture Content and Solubility of the Indicator Films

The SCh–AC40 film had the highest water content, while the SCh–AC20 film had the lowest. As for film solubility, the SCh–A film had the highest value, followed by the SCh–AC20, SCh–AC28, and SCh–AC40 values (Figure 7c,d).

#### 3.8.3. Water Vapor Transmission Rate (WVTR)

The SCh–AC40 film had the lowest WVTR value, while the SCh–AC20 film had the highest (Figure 7e).

### 3.9. Antioxidant Activity of the Indicator Films

The SCh–AC40 film had the highest antioxidant activity value, followed by the SCh–AC28, SCh–AC20, and SCh–A films (Figure 7f).

## 4. Banana Ripening Monitoring

**Figure 8:**
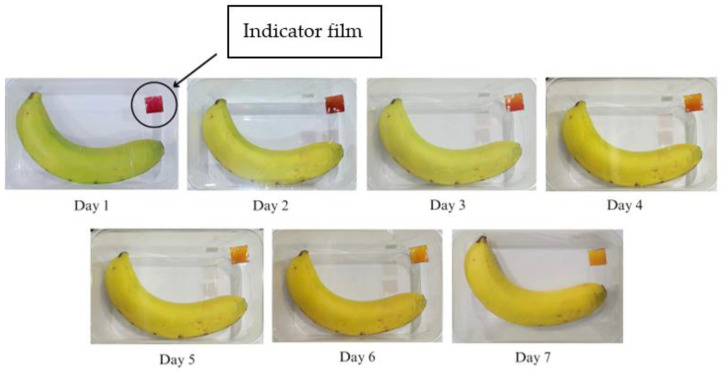
Changes in color of the SCh–A indicator film on banana packaging.

The results of the application of the SCh–A indicator film in monitoring banana ripeness during room temperature storage (25 °C) can be seen in Figure 8. The SCh–A indicator film showed a color change from red to pink–yellow beginning on the 3rd day of storage and another color change to almost entirely yellow on the 6th day of storage.

**Figure 9:**
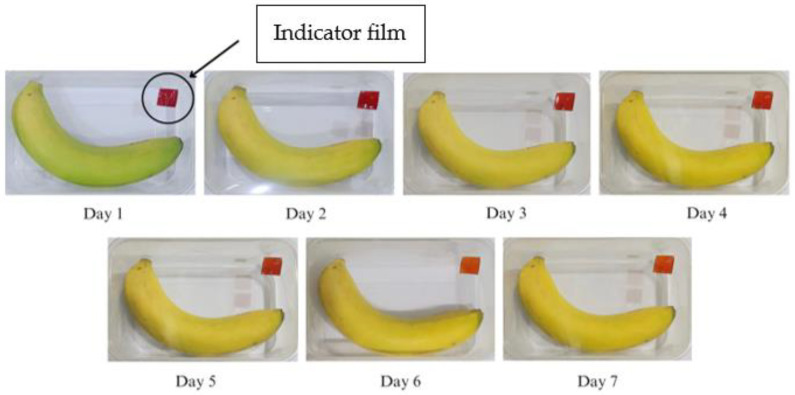
Changes in color of the SCh–AC20 indicator film on banana packaging.

The results of the application of the SCh–AC20 indicator film in monitoring the ripeness of a banana during room temperature storage (25 °C) can be seen in Figure 9. The SCh–AC20 indicator film showed a change in film color from red to pink beginning on the 3rd day of storage and another change into red–yellow on day 5; however, until day 7 of storage, the color of the film did not change completely to yellow.

**Figure 10:**
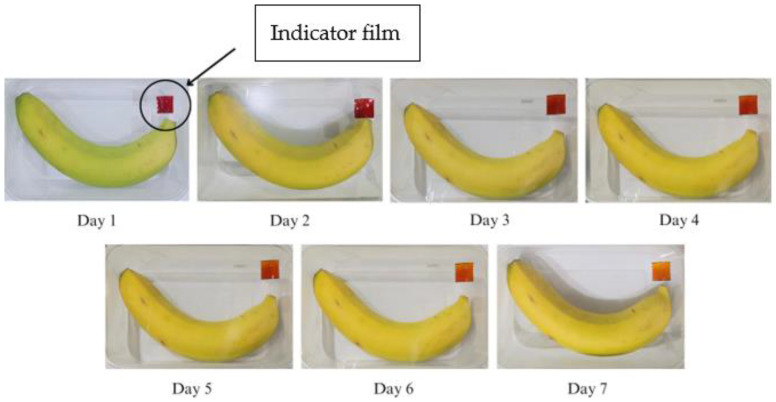
Changes in color of the SCh–AC28 indicator film on banana packaging.

The results of the application of the SCh–AC28 indicator film in monitoring the ripeness of a banana during room temperature storage (25 °C) can be seen in Figure 10. The SCh–AC28 indicator film showed a color change from red to faded red on the 3rd day of storage and another change into red-yellowish on the 4th day, but until the 7th day of storage, the film discoloration only occurred on a portion of the film surface.

**Figure 11:**
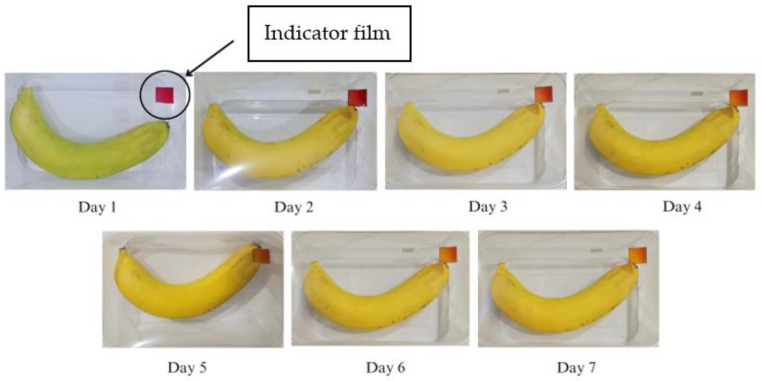
Changes in color of the SCh–AC40 indicator film for banana packaging.

The results of the application of the SCh–AC40 indicator film in monitoring the ripeness of a banana during room temperature storage (25 °C) can be seen in Figure 11. The SCh–AC40 indicator film showed a color change from red to red–yellow on the 3rd day of storage, and on the 7th day of storage, the film color changed almost completely to yellow.

## 5. Discussion

In this study, anthocyanin was contained in red dragon fruit peel at 0.122 ± 0.01 mg/g. Other research similarly discovered anthocyanin in red dragon fruit peel at a level of 0.105 mg/g [15]. The concentration method was applied using a rotary evaporator at 55 °C for the extraction of anthocyanins because anthocyanins are thermolabile compounds. This method was selected in order to prevent anthocyanin damage due to high temperatures, in which case the temperature at which this method was implemented is considered to be the optimal condition for anthocyanin extraction [15].

The anthocyanin extracts obtained were then used as color extracts, and it was determined that two of the color extracts used had the ability to change color depending on the pH conditions. When the color extract without the addition of gambier catechins was used, a significant color change was seen at pH 12 into purple and at pH 13 into yellow. This is in accordance with the research conducted by Apriliyanti et al. [28], which found that at pH 2–10, red dragon fruit peel extract has a reddish-purplish color, which changes into yellow at pH 12–13. In general, anthocyanins will form a pink color at pH 4–5, purple at pH 6–7, and yellow at pH values above 10 [16]. In the color extract added with gambier catechins, a significant color change was obtained at pH 1 to purple. Then, another color change occurred at pH 13 to purple–yellow. The difference in the color series produced by the two types of color extracts was due to the anthocyanin co-pigmentation process rendered by the addition of gambier catechins. Co-pigmentation is a phenomenon of the formation of molecular or complex associations by anthocyanins and co-pigment compounds. The effect of co-pigmentation is manifested as an increase in the color intensity and a change in the color tone of the anthocyanin solution [29]. This phenomenon is known as the hyperchromatic effect (ΔA), which refers to an increase in the intensity of the anthocyanin color, which results in an increase in absorbance and a bathochromic effect (Δλmax), i.e., a shift in the maximum absorbance wavelength (λmax) towards a higher value [30].

Analyses of the surface colors of the indicator films using a colorimeter were performed on the 1st and 14th days after casting. Throughout the storage period, the SCh–A film showed increased L*, a*, and b* values. This indicates a change in the color of the film into brighter red-yellowish. The SCh–AC20 film showed increased L* and b* values, but the a* value decreased. This indicates that the color of the film turned lighter and yellower, but the redness was reduced. The SCh–AC28 film showed decreased L*, a*, and b* values, indicating that the color of the film grew darker, and that the reddish and yellowish colors of the film had decreased toward green and blue. The SCh–AC40 film showed decreased L* and a* values, but the b* value increased. This indicates that the color of the film became darker and yellower, while the redness decreased toward green. Based on the L*, a*, and b* values, the SCh–A film obtained the highest total color difference (Δ*E*), followed by SCh–AC20, SCh–AC28, and SCh–AC40. These results indicate that the most visible discoloration occurred on the SCh–A film, with decreases proportionate to the amount of gambier catechins added. The addition of gambier catechins as a co-pigment can form intramolecular bonds with anthocyanins, which increases the stability [31,32]. Anthocyanin molecules in the form of electron-poor flavylium cations can associate with catechin compounds as electron-rich co-pigments, so that the hydration reaction that changes the anthocyanin structure from colored flavylium cations to colorless chalcones can be prevented [33].

The results of the sensitivity analysis of the SCh–AC40 indicator film after dipping in pH 1–13 buffer showed a color change that was similar to the change that occurred to the color extract. The color change varied slightly, but it was clearly visible at pH 13 as a yellow color. Pramitasari et al. [15] previously found that an indicator film made from the anthocyanin extract of red dragon fruit peel experienced a color change to red–yellow at pH 12 and turned completely yellow at pH 13. This finding bears a similarity with the color change experienced by the SCh–AC40 film, which was analyzed for its sensitivity; therefore, it was concluded that dye based on the anthocyanin of red dragon fruit peel has the ability to change color and has the potential to be used as an indicator film [14].

The results of the SEM morphological analysis showed that the SCh–AC40 film had a heterogeneous structure, as indicated by the presence of highly visible gray and white aggregates. The higher concentration of gambier catechins added increased the amount of sedimentation of insoluble components in the film matrix captured by SEM. This is because gambier catechins are insoluble in cold water and form crystals in dry conditions [34]. The crystalline form of this catechin compound is an aggregate in the film matrix, which induces the formation of a heterogeneous film structure [35]. The results of the FTIR analysis show a similarity with the results of the research by González et al. [24], where the curve shows peaks at wavelengths of 3268 cm^−1^ (O–H stretching), 2926 cm^−1^ (C–H stretching), and 1633 cm^−1^ (O–H bending from water bonds). The band at 1411 cm^−1^ corresponds to CH_2_ bending, the band at 1148 cm^−1^ corresponds to CO deformation, and the band at 1074 cm^−1^ corresponds to COH bending. In addition, the 924 cm^−1^ band is associated with the skeletal mode vibration of α-1,4-glycosidic, namely, the COC bond. The addition of gambier catechins did not cause any new bands, but it caused changes in the absorption area between 1000 and 500 cm^−1^. This may be due to changes in the matrix vibration in CO caused by the addition of gambier catechins [24].

The indicator film thickness analysis results show that the SCh–A film was the thinnest of all the films. This finding is in line with the research by Santoso et al. [34], who reported that the addition of gambier catechins increases the thickness of the film as an added material is used. Gambier catechins, which can form crystals in dry conditions, cause the total solids in the film suspension to increase, so that the thickness of the cast film increases [34]. Based on the results of the analysis of the mechanical properties, the SCh–A film obtained higher TS and EAB values than SCh–AC20 and SCh–AC28. This indicates that the SCh–AC20 and SCh–AC28 films tended to be more brittle and stiffer. As explained by Santoso et al., the addition of gambier catechins can increase the thickness of the film, thereby reducing its tensile strength [34]. In addition, the conformation of the binding between the starch molecular chains and the formation of the double helix structure can hinder the relative displacement between the chains, which results in a decrease in the elongation at break value [36]. The SCh–AC40 film obtained the highest tensile strength and elongation at break values, indicating that it possessed the strongest and most elastic film properties. Chen et al. [36] reported that polyphenols in tea in a certain concentration can increase the film’s tensile and resistance-to-stress properties without reducing its elongation ability. As catechins are natural resins suitable for use as reinforcing agents, they can improve the mechanical properties of the film [37]. In addition, gambier catechins have active hydroxyl groups (-OH); therefore, the higher the concentration of gambier catechins added, the higher the number of OH groups in the film matrix. The OH group can bind to starch molecules and interfere with the rolling and binding between starch molecules, giving it a significant role in increasing the mobility of the film matrix polymer chains, as well as the elasticity and elongation at break value of the film [34,36].

The results of the analysis of film transparency are supported by the results of light transmittance. It was found that the transmittance value of the SCh–AC40 film formed the gentlest curve of all the films, indicating that the SCh–AC40 film had the best ability to inhibit visible light at a wavelength of 380–700 nm. As for transparency, no significant difference was found between the treatments; however, it was found that the transparency value decreased as the concentration of gambier catechins increased, which is in line with the transmittance curve that is formed [29]. In addition, a higher number of solids in the film matrix, as well as greater thickness of the film could prevent light from penetrating the film surface better [28].

Based on the analysis of water content and film solubility, it was found that the SCh–A film had higher water content and solubility than the SCh–AC20, SCh–AC28, and SCh–AC40 films. The addition of gambier catechins could reduce the value of the water content and solubility of the film, which is similar to the findings of Gao et al. [38] that the water content of a film decreases as polyphenols from tea are added to the film. This is because the addition of gambier catechins increases the amount of polymer and the viscosity that forms the film matrix. As the polymer making up the film matrix increases, the total solids also increase, resulting in lower film water content [28]. It should be noted, however, that to a certain extent, the addition of catechins in large quantities can cause a resurgence in the water content of the film. When catechins interact with anthocyanins or other polymers, there will be free catechins, which causes an increase in the availability of OH groups. Based on the results of the analysis of film solubility, the higher the concentration of catechins, the lower the solubility value of the film [34]. This is because gambier catechins are difficult to dissolve in cold water.

According to the WVTR analysis results, the SCh–AC40 film had the lowest WVTR value of all the films, indicating that the SCh–AC40 film had the best ability as a water vapor barrier. This is in accordance with Ku et al.’s finding that the addition of catechins in the film matrix can reduce the WVTR value of the film. This could be because the addition of catechins causes changes in matrix interactions and modifications in the structure of the film formed [39].

From the antioxidant activity test results, it was found that the SCh–A film had the lowest antioxidant activity of all the films. In the SCh–A film, the antioxidant activity comes from chitosan and anthocyanin compounds. Anthocyanins, which are polyphenolic compounds, can act as antioxidants, while chitosan can prevent the initial stage of oxidation through the interaction between the free NH_2_ group in the C_2_ position of chitosan, which binds to the H^+^ group from the film solution to form NH_3_^+^. This compound will then interact with the DPPH free radical to form a stable molecule [40]. Chen et al. previously reported that the addition of tea polyphenols, which is comparable to the gambier catechins in the case of this research, increased the antioxidant activity of the film [36]. According to research by Anggraini et al. [41], gambier catechins contain many polyphenolic compounds belonging to the flavonoid group, which act as good antioxidant compounds. Catechins can donate hydrogen atoms or their single electrons to radical compounds, which causes oxidation–reduction reactions to occur, so that radical compounds that initially had unpaired electrons now have their electrons paired and thus become non-radical [42].

The SCh–A, SCh–AC20, SCh–AC28, and SCh–AC40 films all showed color changes from red to dull red or red–yellow on the 3rd day of storage. This is in line with the research of Ardiyansyah et al., which reported an increase in pH on the 4th day of storage, indicating that the bananas were starting to ripen. The ripening process of bananas is marked by increases in the texture value, pH, and CO_2_ gas produced and by a decrease in the vitamin C level in the fruit. The color change occurring on the indicator films is due to respiration or metabolism that takes place during the fruit ripening process, as well as the activity of enzymes and microorganisms during the fruit decomposition process. This process causes an increase in the pH of the environment to become alkaline, resulting in a change in the color of the indicator film to yellow [43]. In addition, this can be caused by the presence of other compounds, namely, betalain color pigments, which consist of betacyanin, a red–purple pigment, and betaxanthin, a yellow–orange pigment. Betalain pigments are stable under acidic pH conditions. As the pH increases, the structure of betalain can be degraded into colorless cyclo-DOPA 5-O-(malonyl)-β-glucoside and yellow betaxanthin [44].

## 6. Conclusions

The addition of gambier catechins to the anthocyanin color extract of red dragon fruit peel could successfully increase the color stability of the film without reducing the film’s ability to change under certain pH conditions. The SCh–AC40 indicator film produced the best color stability, mechanical properties, light and water vapor barrier ability, and antioxidant activity. When applied to banana packaging, the SCh–AC40 indicator film showed a color change that was easily observable during storage, supported by changes in the environmental pH toward an alkaline condition. Based on the results obtained, the SCh–AC40 indicator film has the most potential to be developed as smart packaging for banana ripeness detection.

## Figures and Tables

**Table 1:** Color extract formulation.

Sample	Anthocyanins:Gambier Catechins (*w*/*w*)
SCh–A	1:0
SCh–AC20	1:20
SCh–AC28	1:28
SCh–AC40	1:40

**Table 2:** Composition of indicator film solution per print unit (20 mL).

Composition	Amount (mL)
1% (*w*/*v*) chitosan solution	7.46
2% (*w*/*v*) cassava starch solution	7.46
85% glycerol	0.08
Color extract	5

**Table 3:** The results of the surface color analysis of the indicator films using a colorimeter.

Sample	1st Day			14th Day			
	L*	a*	b*	L*	a*	b*	Δ*E*
SCh–A	32.50 ± 1.10 ^a^	35.62 ± 3.51 ^a^	14.45 ± 3.25 ^a^	34.87 ± 1.65 ^bc^	38.59 ± 1.10 ^b^	19.36 ± 2.14 ^b^	8.09 ± 5.29 ^a^
SCh–AC20	34.13 ± 2.16 ^a^	41.16 ± 6.73 ^a^	12.25 ± 3.02 ^a^	36.51 ± 0.86 ^c^	39.51 ± 0.90 ^b^	19.25 ± 0.92 ^b^	7.83 ± 1.90 ^a^
SCh–AC28	34.99 ± 0.65 ^a^	39.92 ± 4.14 ^a^	15.1 ± 1.65 ^a^	32.83 ± 0.20 ^ab^	37.29 ± 0.12 ^b^	14.72 ± 0.09 ^a^	4.43 ± 3.34 ^a^
SCh–AC40	31.67 ± 0.16 ^a^	33.32 ± 0.28 ^a^	11.07 ± 0.21 ^a^	31.41 ± 0.22 ^a^	31.09 ± 0.63 ^a^	11.46 ± 0.42 ^a^	2.33 ± 0.57 ^a^

All data are presented as means ± standard deviations. Different letters in each column indicate a real difference; α ≤ 0.05.

**Table 4:** Thickness and mechanical properties of the indicator film.

Films	Thickness (mm)	Tensile Strength (MPa)	Elongation at Break (%)
SCh–A	0.13 ± 0.00 ^a^	0.19 ± 0.02 ^b^	2.22 ± 0.35 ^b^
SCh–AC20	0.15 ± 0.01 ^b^	0.12 ± 0.03 ^a^	1.29 ± 0.01 ^a^
SCh–AC28	0.15 ± 0.00 ^b^	0.12 ± 0.01 ^a^	1.42 ± 0.24 ^a^
SCh–AC40	0.14 ± 0.00 ^ab^	0.23 ± 0.02 ^b^	4.88 ± 0.27 ^c^

All data are presented as means ± standard deviations. Different letters in each column indicate a real difference; α ≤ 0.05.

## Data Availability

The data presented in this study are available on request from the corresponding author.
